# Supplementary material for: Complexome profiling of the Chlamydomonas psb28 mutant reveals TEF5 as an early PSII assembly factor
Source: Plant Cell. 2025 Mar 17;37(6):koaf055. doi: 10.1093/plcell/koaf055 (PMC12164590; doi:10.1093/plcell/koaf055)
Supplement: koaf055_Supplementary_Data [file koaf055_supplementary_data.zip › psb28_interactive.zip!/plots/intensity/qualityPlots/peptides/NUO11_Cre09.g402552.t1.1.html]

### Cre09.g402552.t1.1

Trivial name: NUO11  
SeqCoverage [median]: 5.30  
Mass: 24.24 kDa  
  
 

| Sequence | MissedCleavage | Mass [Da] | wtIntensity | muIntensity | Score |
| --- | --- | --- | --- | --- | --- |
| SATDVEALNMLK | 0 | 1290.65 | 13797.18 | 21895.35 | 225.66 |
|  |  |  | 13797.2 | 21895.3 |  |
